# Long Use of Tracheotomy Tube

**Published:** 1913-02

**Authors:** 


Long Use of Tracheotomy Tube.—Thomson, in the Proceedings of the Royal Society of Medicine, reported a woman who died at 81. Altogether, she had worn a tracheotomy tube, off and on, for 50 years. A single tube was worn for 16 years and often the tube was not changed for two years. No greater tendency to bronchitis was noted than in most old persons.

